# Preoperative and follow-up variations of psoas major muscle are related to S1 screw loosening in patients with degenerative lumbar spinal stenosis

**DOI:** 10.1186/s12891-024-07298-0

**Published:** 2024-05-28

**Authors:** Siyu Zhou, Fei Xu, Zhuoran Sun, Shuai Jiang, Zhuofu Li, Gengyu Han, Weishi Li

**Affiliations:** 1 Orthopaedic Department, Peking University Third Hospital, No. 49 North Garden Road, Haidian District, Beijing, 100191 China; 2 Peking University Health Science Center, No. 38 Xueyuan Road, Haidian District, Beijing, 100191 China; 3 Beijing Key Laboratory of Spinal Disease Research, Beijing, China; 4 Engineering Research Center of Bone and Joint Precision Medicine, Ministry of Education, Beijing, China

**Keywords:** Degenerative lumbar spinal stenosis, First sacral vertebra, Pedicle screw loosening, Psoas major muscle, Paraspinal muscles, Osteoporosis

## Abstract

**Background:**

It was reported the paraspinal muscle played an important role in spinal stability. The preoperative paraspinal muscle was related to S1 screw loosening. But the relationship between preoperative and postoperative change of psoas major muscle (PS) and S1 pedicle screw loosening in degenerative lumbar spinal stenosis (DLSS) patients has not been reported. This study investigated the effects of preoperative and follow-up variations in the psoas major muscle (PS) on the first sacral vertebra (S1) screw loosening in patients with DLSS.

**Methods:**

212 patients with DLSS who underwent lumbar surgery were included. The patients were divided into the S1 screw loosening group and the S1 screw non-loosening group. Muscle parameters were measured preoperatively and at last follow-up magnetic resonance imaging. A logistic regression analysis was performed to investigate the risk factors for S1 screw loosening.

**Results:**

The S1 screw loosening rate was 36.32% (77/212). The relative total cross-sectional areas and relative functional cross-sectional areas (rfCSAs) of the PS at L2–S1 were significantly higher after surgery. The increased rfCSA values of the PS at L3–S1 in the S1 screw non-loosening group were significantly higher than those in the S1 screw loosening group. The regression analysis showed male, lower CT value of L1 and longer segment fusion were independent risk factors for S1 screw loosening, and postoperative hypertrophy of the PS was a protective factor for S1 screw loosening.

**Conclusions:**

Compared to the preoperative muscle, the PS size increased and fatty infiltration decreased after surgery from L2–3 to L5–S1 in patients with DLSS after short-segment lumbar fusion surgery. Postoperative hypertrophy of the PS might be considered as a protective factor for S1 screw loosening. MRI morphometric parameters and postoperative selected exercise of PS for DLSS patients after posterior lumbar fusion surgery might contribute to improvement of surgical outcome.

## Introduction

Surgery for degenerative lumbar diseases usually involves the first sacral vertebra (S1) at the fused caudal level as the L5–S1 level presents the highest prevalence of disc degeneration and may require decompression and fusion interventions [1]. Loosening of the S1 pedicle screw is a common complication [1], with the reported rates being 15.6–41.9% [2–4]. Furthermore, severe pedicle screw loosening may cause back pain and require revision surgery [5, 6].

Age, osteoporosis, long-segment fusion, and degenerative preoperative paraspinal muscles are reported as risk factors for S1 screw loosening [7–9]. The paraspinal muscle is also crucial to maintaining spinal stability, and paraspinal muscle degeneration is associated with surgical complications and clinical outcomes [10–14]. Some reports have focused on the relationship between paraspinal muscle degeneration and screw loosening [3, 15]. The cross-sectional areas (CSA) of the multifidus and erector spinae muscles on preoperative magnetic resonance imaging (MRI) have been reported as risk factors for S1 screw loosening [7]. But after posterior lumbar fusion surgery, extensor paraspinal muscles have been destroyed with decreased CSA and increased fat infiltration [16], emphasizing the importance of psoas muscle. Previous studies report that spinal stabilization exercises focusing on the major psoas muscle (PS) play a significant role in reducing lower back pain and improving functional outcomes [17, 18]. However, the relationship between pre- and postoperative variations of paraspinal muscles and S1 screw loosening has not been well documented.

This study was designed to investigate the relationship between pre- and postoperative changes in PS morphology and S1 screw loosening. We hypothesized that the changes of PS morphology might be related to S1 screw loosening.

## Methods

### Patients

Medical records of patients who underwent spinal fusion operations including L5–S1 level for degenerative lumbar spinal stenosis (DLSS) were retrospectively reviewed from 2015 to 2018 at a single hospital. This study was approved by the Ethics Review Board of this institution. The inclusion criteria were as follows: (1) 2 or 3 fused segments; (2) lowest instrumented vertebra (LIV) at S1; and (3) minimum 1-year follow-up. The exclusion criteria included: (1) evidence of scoliosis, including idiopathic scoliosis, congenital scoliosis, traumatic scoliosis, ankylosing spondylitis, or presence of tuberculosis or tumors; (2) history of spinal surgery; (3) lack of preoperative and follow-up MRI; (4) application of iliac screws; and (5) application of cement-augmented pedicle screws. The patients were divided into two groups according to the S1 screw status at the final follow-up: S1 screw loosening (77 patients) group and controls (no evidence of S1 screw loosening) (135 patients). According to Gpower 3.1, when the power value was 0.95 and effect size was 0.5, the sample size was 210. And in our study, the sample size was 212, which was larger than 210. So the power value of our study was > 0.95. The surgical procedures were as follows: Paraspinal muscles were dissected away from posterior elements (spinal process, lamina, and facet joints), and no special muscle preservation technique was used. Then laminotomy was performed at the surgical level with preservation of the adjacent supraspinatus and interspinous ligaments. The patients underwent laminotomy, lumbar fusion, and fixation with a transpedicular screw fixation device. The pedicle screws used were all conventional screws and the monocortical fixation method was used for all screws. All surgeries were performed using a consistent technique by senior chief physicians with similar experience.

### Radiographic assessment

**Fig. 1:**
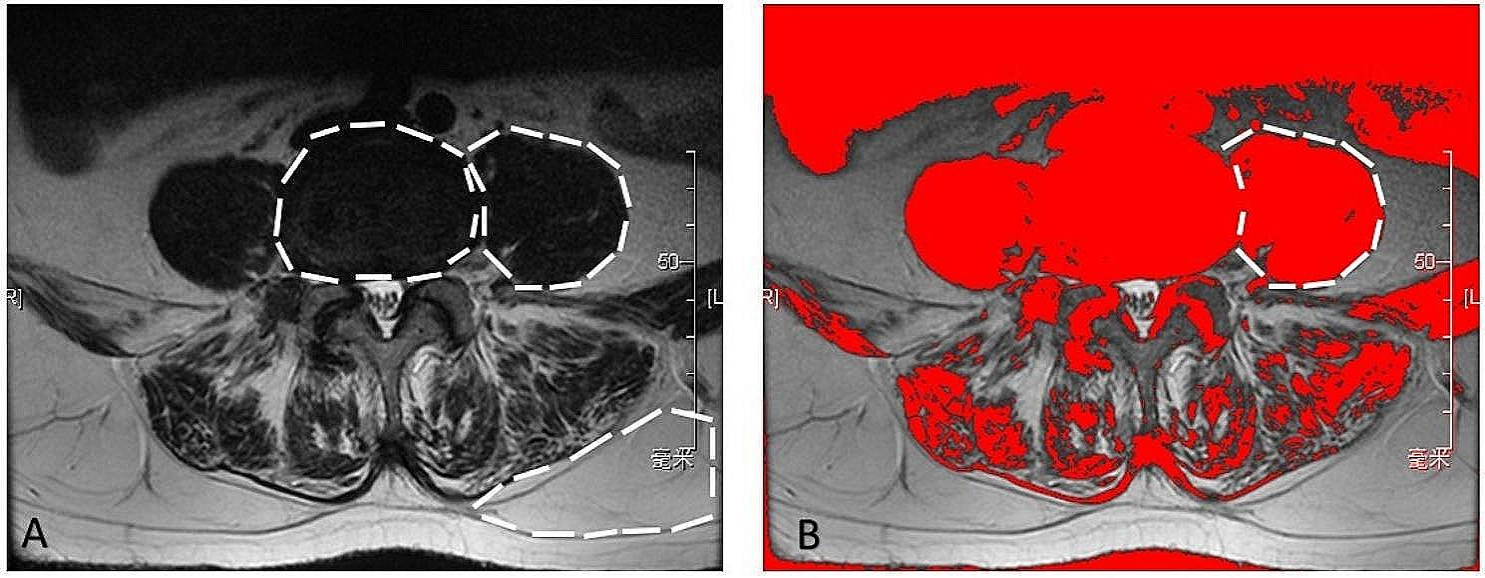
Measurements of paraspinal muscle parameters on magnetic resonance imaging. The regions of the intervertebral disc, psoas major muscle (PS), and subcutaneous fat are outlined by white lines. For the psoas major muscle, the total and functional muscles are outlined on the right and left sides, respectively. The subcutaneous fat is outlined on the left side by black lines. rtCSA = tCSA/ disc area (at the same level). rfCSA = fCSA/ disc area (at the same level). changed rtCSA = postoperative rtCSA/preoperative rtCSA. changed rfCSA = postoperative rfCSA/preoperative rfCSA. FI=(tCSA-fCSA)/tCSA. MFI = muscle index/ subcutaneous fat index. Changed rtCSA and rfCSA > 1 meant CSA increased after surgery and changed rtCSA and rfCSA < 1 meant CSA decreased after surgery

All muscle parameters were measured using Image J software (version 1.52, National Institutes of Health, Bethesda, Maryland, USA) on lumbar MRI performed before surgery and repeated at the last follow-up (at least 1 year) after surgery. The functional muscle area was measured using threshold techniques [19, 20]. The plane parallel to the corresponding middle intervertebral disc was selected for measurements. PS parameters at the different levels of intervertebral discs on T2-weighed fast spin echo sequences were measured. The regions of interest (ROI) were defined by outlining the target muscles [3] and used to compute the CSA and mean T2 signal intensity. The following regions were measured in each image: total PS (including functional muscle, intramuscular fat, and soft tissue), functional PS, cross section of intervertebral disc, and subcutaneous fat (Fig. 1).

**Fig. 2:**
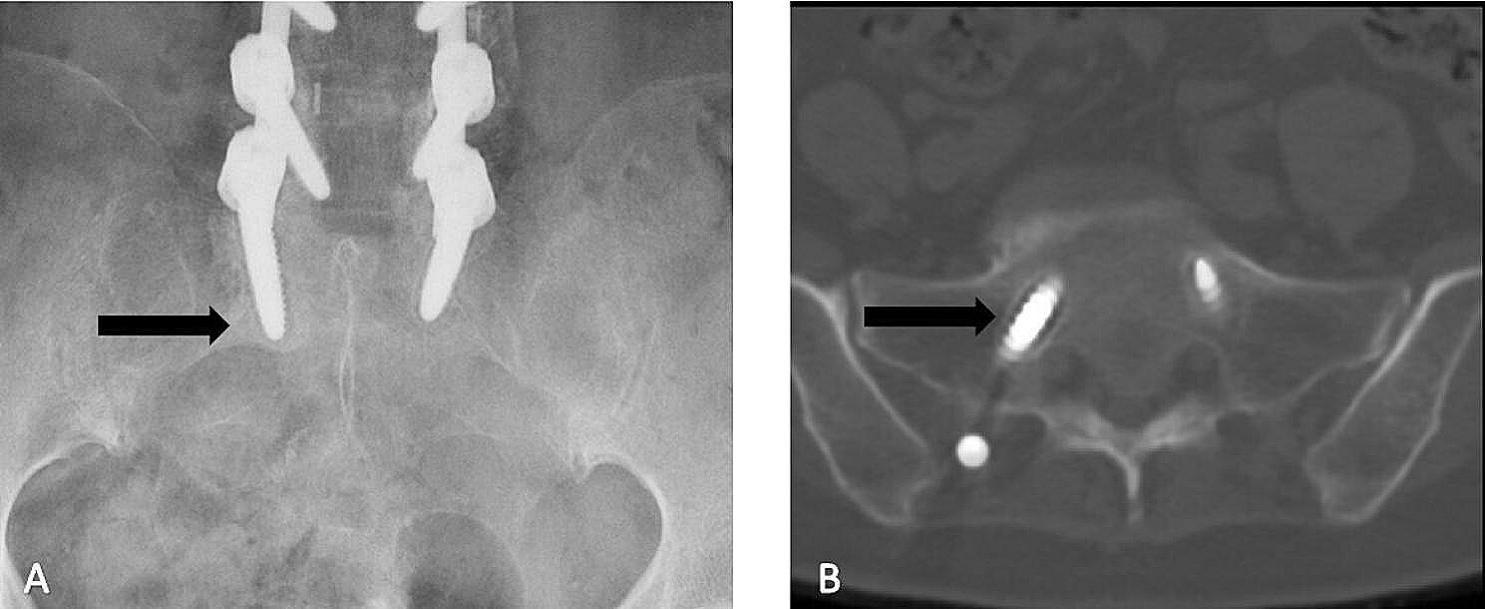
A halo sign surrounding the first sacral vertebra (S1) pedicle screw on radiographs. (**A**) The radiolucent line on a plain radiograph is indicated by a black arrow. (**B**) The radiolucent line on the computed tomography image is indicated by a black arrow

To reduce the effects of different heights and weights on muscle parameters, values for relative muscle CSA (rCSA), defined as the ratio of CSA to disc area at the same level, the relative total CSA (rtCSA), and relative functional CSA (rfCSA) were used. The change in rtCSA was calculated as postoperative rtCSA/preoperative rtCSA and the change in rfCSA was calculated as postoperative rfCSA/preoperative rfCSA. Furthermore, FI was calculated as (tCSA-fCSA)/tCSA. The ratio of the T2 signal intensity of the muscle to that of subcutaneous fat at the same level was used as the muscle-fat index (MFI) [21, 22]. Besides, the preoperative MF and ES muscular parameters at L4-5 were evaluated [23, 24]. S1 screw loosening was independently evaluated from anteroposterior and lateral films (Discovery XR650 machine, General Electric Company) or from computed tomography (CT) images (Revolution CT, General Electric Company) at the final follow-up by a single observer blinded to the clinical information, and the evaluation of S1 screw loosening was separated from the muscle measurements. The intra- and interclass correlation coefficient (ICC) was calculated to determine inter- and intra-observer reliability using SPSS (version 23.0, IBM). S1 screw loosening was defined as a halo sign with a circumferential radiolucent line of ≥ 1 mm around the S1 pedicle screw (Fig. 2) [25, 26].

Sagittal spinopelvic parameters were measured using standing X-rays before surgery and at the early postoperative period and included lumbar lordosis (LL, defined as the angle between the upper endplate of L1 and the sacral plate), the sacral slope (SS, defined as the angle between the horizontal line and the sacral plate), the pelvic incidence (PI, defined as the angle between the perpendicular from the midpoint of the upper endplate of S1 and a line connecting the center of the femoral head to the center of the upper endplate of S1), and the pelvic tilt (PT, defined as the angle between the vertical and the line through the midpoint of the sacral plate to the femoral head axis). And L4-S1 angulation was defined as the angle between the upper endplate of L4 and the sacral plate. Preoperative C7 sagittal vertical axis (SVA) was defined as the horizontal distance between a plumb line drawn from the center of C7 and the plumb line from the posterior-superior corner of the sacrum. Preoperative computerized tomography (CT) was performed, and a threshold of Hounsfield Units (HU) value ≤ 110 was used to judge osteoporosis based on CT scan [27, 28]. The clinical outcome was evaluated at last follow-up by using Oswestry Disability Index (ODI) and Visual Analogue Scale (VAS).

### Statistical analysis

Statistical analysis was conducted with SPSS Statistics for Windows version 23.0 (IBM Corp). The paired-sample t-test was used to compare differences in preoperative and follow-up PS parameters. Clinical information and radiographic parameters were compared between the S1 screw loosening and S1 screw non-loosening groups. These parameters were compared between the clinical and radiological S1 screw loosening groups. Student’s t-test, a non-parametric test (for continuous data), and the χ2 test (for categorical data) were conducted to determine the statistical difference. A binary logistic regression model was used to identify independent risk factors for S1 screw loosening. Receiver operating characteristic (ROC) curve analysis was performed to determine the cutoff value. Statistical significance was set at a P*-*value < 0.05.

## Results

Overall, 212 patients were enrolled; the mean age was 58.51 ± 9.67 (range, 29–80) years. This study included 101 male and 111 female patients. The mean follow-up time was 27.90 ± 11.54 (range, 12–42) months. Of the included patients, 59 (27.83%) underwent L3–S1 fusion and 153 (72.17%) underwent L4–S1 fusion. The ICC for intra-observer agreement in CSA of PS was 0.900 (95% confidence interval [CI], 0.788–0.954; *P* < 0.001). The ICC for inter-observer agreement in CSA of PS was 0.933 (95% confidence interval [CI], 0.812–0.977; *P* < 0.001).

According to the paired-sample t-test (Table 1), the rtCSAs and rfCSAs of the PS at L2–3, L3–4, L4–5, and L5–S1 were higher on follow-up MRI than on preoperative MRI (*P* < 0.05). In addition, the FIs of the PS at L4–5 and L5–S1 were lower on follow-up MRI than on preoperative MRI (*P* < 0.05). Moreover, the preoperative rtCSA and rfCSA of the PS gradually increased from the cephalic lumbar spine to the caudal lumbar spine (*P* < 0.05). Changes in the rtCSA and rfCSA of the PS at follow-up were similar to those in the preoperative PS values.

**Table 1:** Comparison of preoperative PS and follow-up PS

	Preoperative PS (%)	Follow-up PS (%)	P value
L2-3 rtCSA	30.81 ± 12.99	34.21 ± 12.65	< 0.001**
L2-3 rfCSA	28.32 ± 12.32	31.56 ± 12.14	< 0.001**
L2-3 FI	8.36 ± 6.54	8.04 ± 4.53	0.673
L2-3 MFI	19.62 ± 12.21	19.75 ± 5.13	0.912
L3-4 rtCSA	50.48 ± 18.17	56.33 ± 18.82	< 0.001**
L3-4 rfCSA	46.93 ± 17.62	53.38 ± 18.82	< 0.001**
L3-4 FI	6.70 ± 9.86	4.81 ± 3.03	0.007**
L3-4 MFI	17.48 ± 9.38	17.74 ± 4.53	0.714
L4-5 rtCSA	64.09 ± 22.54	72.03 ± 22.60	< 0.001**
L4-5 rfCSA	59.83 ± 21.64	69.18 ± 22.24	< 0.001**
L4-5 FI	6.69 ± 5.77	4.14 ± 2.83	< 0.001**
L4-5 MFI	16.06 ± 6.22	17.00 ± 5.06	0.074
L5-S1 rtCSA	61.09 ± 23.57	67.29 ± 20.58	< 0.001**
L5-S1 rfCSA	57.60 ± 22.77	64.81 ± 20.07	< 0.001**
L5-S1 FI	5.94 ± 7.74	3.73 ± 2.46	< 0.001**
L5-S1 MFI	17.02 ± 11.05	17.33 ± 5.67	0.701

Mean values were presented as ± standard deviation

PS, psoas muscle; rtCSA, relative total cross-sectional area; rfCSA, relative functional cross-sectional area; FI, fatty infiltration; MFI, muscle-fat index

*P < 0.05

**P < 0.01

At the final follow-up, 36.32% of the patients experienced S1 screw loosening (Table 2), which included more male patients than those in the control group (58.44% vs. 41.48%, *P* < 0.05). Compared to the S1 screw loosening group, the control group had fewer fused levels (2.36 ± 0.48 vs. 2.23 ± 0.42, *P* < 0.05). Regarding changes in the preoperative and follow-up PS, the increased rfCSA values at L3–4, L4–5, and L5–S1 in the control group were higher than those in the S1 screw loosening group (1.21 ± 0.29 vs. 1.14 ± 0.36, 1.23 ± 0.22 vs. 1.14 ± 0.23, 1.53 ± 2.86 vs. 1.13 ± 0.26; *P* < 0.05 for each). Moreover, changes in the rtCSA at the L5–S1 were higher than those observed in the S1 screw loosening group (1.29 ± 1.11 vs. 1.10 ± 0.26, *P* < 0.05). Pre- and postoperative spinopelvic parameters including SS, PT, LL, PI, PI-LL and L4-S1 angulation were similar between groups.

**Table 2:** Comparison of demographic, changed PS parameters (follow-up and preoperative) and postoperative spinopelvic parameters between the S1 screw non-loosening group and S1 screw loosening group

	S1 screw non-loosening group	S1 screw loosening group	P value
Number	63.68%(135/212)	36.32%(77/212)	
Age (years old)	58.19 ± 9.74	59.08 ± 9.59	0.523
Gender (Male/Female)	56/79	45/32	0.022*
BMI(kg/m^2^)	26.04 ± 4.14	26.81 ± 4.71	0.211
Subcutaneous fat index at L4-5	160.60 ± 32.88	166.96 ± 36.93	0.212
CT value of L1	157.35± 45.64	140.17± 43.05	0.008*
Fused levels	2.23 ± 0.42	2.36 ± 0.48	0.037*
L5-S1 intervertebral fusion	85.9% (116/135)	76.6% (59/77)	0.094
Diabetes (Yes/No)	14/63(18.2%)	16/119(11.9%)	0.223
Smoking status (Yes/No)	13/64(16.9%)	21/114(15.6%)	0.847
Follow-up time (months)	28.10 ± 12.57	27.53 ± 9.53	0.710
Follow-up ODI	7.67 ± 7.89	8.59 ± 9.77	0.466
Follow-up VAS (back)	1.06 ± 1.49	1.32 ± 1.71	0.269
Follow-up VAS (leg)	1.02 ± 1.71	0.68 ± 1.37	0.147
Psoas major muscular parameters			
L2-3 changed rtCSA	1.15 ± 0.28	1.18 ± 0.37	0.894
L2-3 changed rfCSA	1.18 ± 0.29	1.16 ± 0.34	0.652
L2-3 changed MFI	1.10 ± 0.39	1.22 ± 0.42	0.142
L3-4 changed rtCSA	1.18 ± 0.29	1.13 ± 0.32	0.067
L3-4 changed rfCSA	1.21 ± 0.29	1.14 ± 0.36	0.030*
L3-4 changed MFI	1.13 ± 0.46	1.17 ± 0.39	0.415
L4-5 changed rtCSA	1.18 ± 0.21	1.12 ± 0.24	0.062
L4-5 changed rfCSA	1.23 ± 0.22	1.14 ± 0.23	0.007**
L4-5 changed MFI	1.13 ± 0.46	1.22 ± 0.51	0.396
L5-S1 changed rtCSA	1.29 ± 1.11	1.10 ± 0.26	0.005**
L5-S1 changed rfCSA	1.53 ± 2.86	1.13 ± 0.26	0.007**
L5-S1 changed MFI	1.15 ± 0.48	1.26 ± 0.59	0.301
Preoperative multifidus and erector spinae muscular parameters at L4-5			
rtCSA of MF at L4-5	0.46 ± 0.19	0.48 ± 0.14	0.465
rfCSA of MF at L4-5	0.32 ± 0.14	0.34 ± 0.10	0.212
FI of MF at L4-5	0.31 ± 0.11	0.28 ± 0.11	0.155
rtCSA of ES at L4-5	0.79 ± 0.28	0.87 ± 0.33	0.158
rfCSA of ES at L4-5	0.59 ± 0.24	0.67 ± 0.27	0.091
FI of ES at L4-5	0.25 ± 0.10	0.23 ± 0.08	0.105
Preoperative spinopelvic parameters			
LL(°)	29.62 ± 11.29	30.53 ± 14.40	0.611
PI(°)	46.86 ± 9.13	46.98 ± 10.27	0.931
PT (°)	18.42 ± 7.49	18.55 ± 10.23	0.920
SS(°)	24.60 ± 7.48	26.24 ± 9.11	0.150
PI-LL(°)	12.93 ± 9.74	11.14 ± 10.03	0.204
L4-S1 angulation	21.07 ± 9.33	21.68 ± 9.73	0.649
SVA(mm)	35.48 ± 42.08	38.76 ± 49.57	0.705
Postoperative spinopelvic parameters			
LL(°)	33.93 ± 8.62	35.85 ± 10.76	0.158
PI(°)	42.99 ± 8.31	44.79 ± 11.27	0.186
PT (°)	18.36 ± 7.58	17.06 ± 7.85	0.238
SS(°)	28.50 ± 6.85	29.92 ± 8.71	0.223
PI-LL(°)	13.37 ± 11.20	14.26 ± 14.94	0.625
L4-S1 angulation	26.53 ± 6.97	27.05 ± 7.56	0.616
Changed pre- and postoperative LL(°)	4.31 ± 9.22	5.31 ± 11.02	0.502

Mean values were presented as ± standard deviation

PS, psoas muscle; MF, multifidus muscle; ES, erector spinae muscle; BMI, indicates body mass index; ODI, Oswestry Disability Index; rtCSA, relative total cross-sectional area; rfCSA, relative functional cross-sectional area; FI, fatty infiltration; MFI, muscle-fat index; SS, sacral slope; PT, indicates pelvic tilt; LL, indicates lumbar lordosis; PI, pelvic incidence; PI-LL, pelvic incidence minus lumbar lordosis; SVA, C7 sagittal vertical axis

Changed rtCSA = follow-up rtCSA/preoperative rtCSA; changed rfCSA = follow-up rfCSA/preoperative rfCSA; changed MFI = follow-up MFI /preoperative MFI

Changed parameters (rtCSA and rfCSA) > 1 meant CSA increased after surgery and changed parameters (rtCSA and rfCSA) < 1 meant CSA decreased after surgery

*P < 0.05

**P < 0.01

**Fig. 3:**
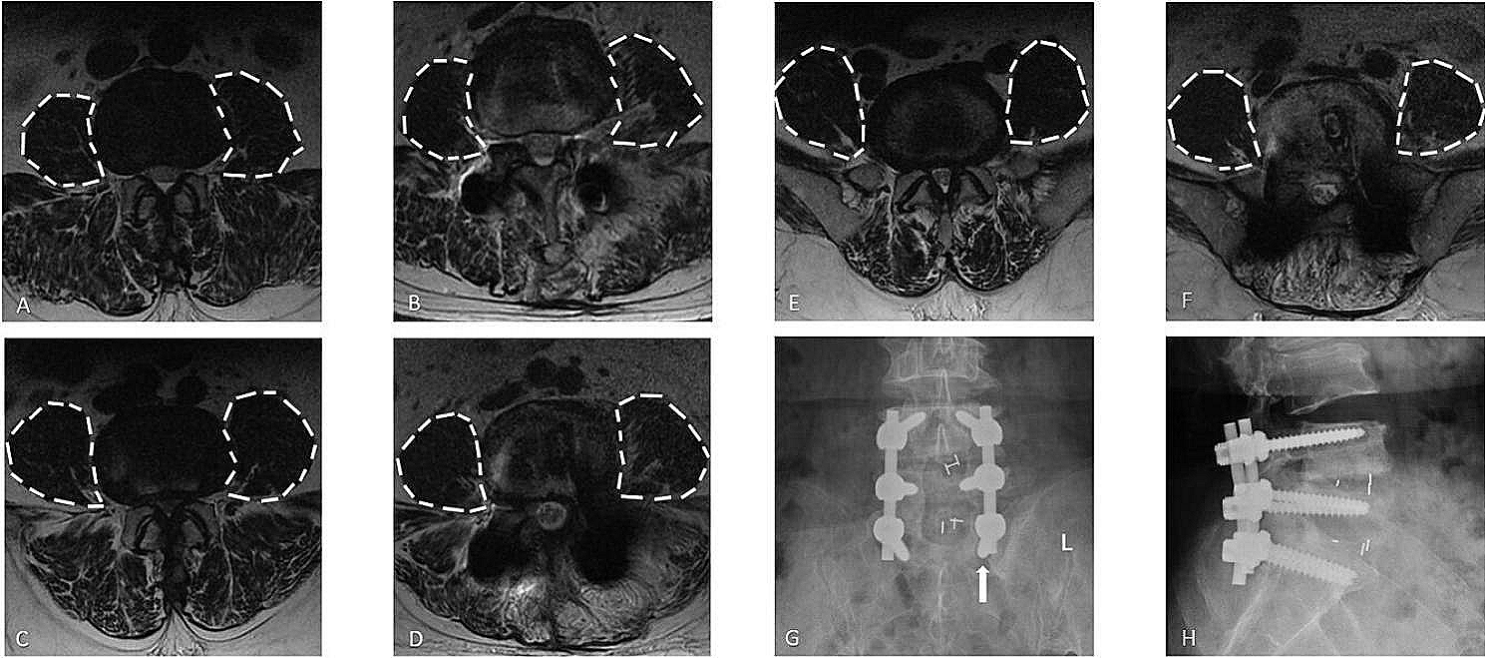
Preoperative and postoperative radiographs of a 59-year-old woman presenting with first sacral vertebra (S1) screw loosening at the 30-month follow-up (**G**, **H**). Preoperative functional cross-sectional areas (fCSAs) of the psoas major muscle (PS) at L3–4 (A), L4–5 (**C**), and L5–S1 (**E**) are shown. Postoperative fCSAs of the PS at L3–4 (**B**), L4–5 (**D**), and L5–S1 (**F**) are shown. Moreover, the ratio of postoperative and preoperative rfCSA at L3-S1 was calculated as 0.89, which was lower than the proposed 1.075 cutoff value

**Fig. 4:**
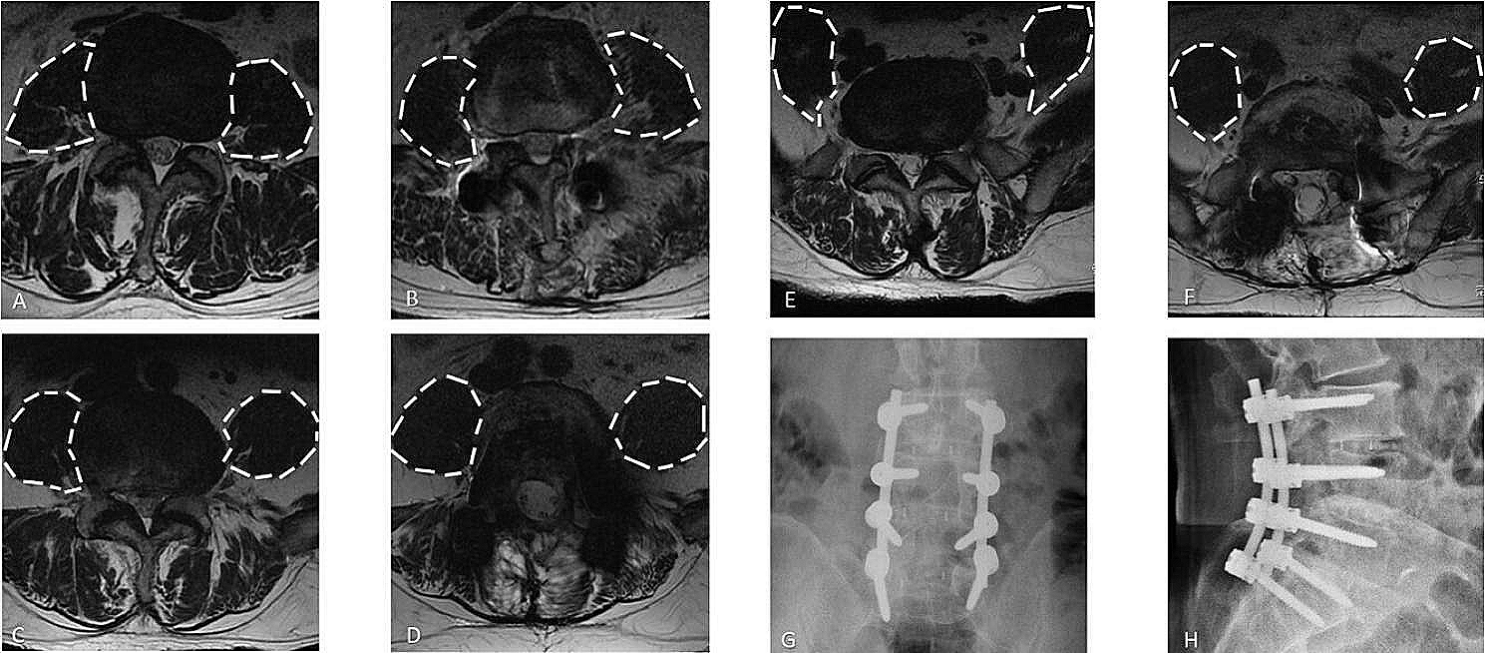
Preoperative and postoperative radiographs of a 69-year-old man presenting with first sacral vertebra (S1) screw non-loosening at the 38-month follow-up (**G**, **H**). Preoperative functional cross-sectional areas (fCSAs) of the psoas major muscle (PS) at L3–4 (A), L4–5 (**C**), and L5–S1 (**E**) are shown. Postoperative fCSAs of the PS at L3–4 (**B**), L4–5 (D), and L5–S1 (**F**) are shown. The ratio of postoperative and preoperative rfCSA at L3-S1 was 1.50, which was higher than the proposed 1.075 cutoff value

Binary logistic regression analysis (Table 3) was performed and included basic factors and potential risk factors with a P-value < 0.05 (age, gender, CT value of L1, fused levels, L5–S1 intervertebral fusion, and the mean changed rfCSA of the PS on L3–4, L4–5, and L5–S1 slices [206 patients had complete PS parameters in three slices]). The regression analysis revealed that male, lower CT value of L1 and longer-segment fusion were independent risk factors for S1 screw loosening, and postoperative compensatory hypertrophy of the PS at L3–4, L4–5, and L5–S1 was a protective factor for S1 screw loosening. A ROC curve was plotted to evaluate the mean changed rfCSA of the PS on L3–4, L4–5, and L5–S1 and S1 screw loosening (Table 4), and the Youden index was 1.075 (Figs. 3 and 4).

**Table 3:** Independent risk factors of S1 screw loosening identified by logistic regression

	OR	95% CI for OR	P value
Age	0.973	0.933–1.013	0.183
Gender	0.465	0.244–0.887	0.020*
CT value of L1	0.985	0.977–0.994	0.001*
Fused levels	3.075	1.430–6.612	0.004*
L5-S1 intervertebral fusion	0.723	0.314–1.665	0.445
Mean changed rfCSA of L3-S1	0.055	0.009–0.329	0.001*

rfCSA, relative functional cross-sectional area

*P < 0.05

**P < 0.01

**Table 4:** Youden Index of mean changed rfCSA of PS at L3-4, L4-5, L5-S1 for S1screw loosening

	S1 screw non-loosening group	S1 screw loosening group	P value
Mean changed rfCSA < 1.075	24.43% (32/131)	49.33% (37/75)	< 0.001**
Mean changed rfCSA ≥ 1.075	75.57% (99/131)	50.67% (38/75)	

PS, psoas muscle; rfCSA, relative functional cross-sectional area

Changed rfCSA = follow-up rfCSA/preoperative rfCSA

Changed rfCSA > 1 meant CSA increased after surgery and changed rfCSA < 1 meant CSA decreased after surgery

*P < 0.05

**P < 0.01

## Discussion

In asymptomatic individuals, the PS reportedly exhibits an increase in size from the cephalic to the caudal part at lumbar levels [29–32]. And this may be related to additional stress would be placed on L4–5 since it commonly occurs at the apex of the lordosis or in the vicinity [33]. In our study, the segmental distribution of the PS in the preoperative patients increased from L2-3 to L4-5, which was consistent with previous research. Besides, in the present study, compared to preoperative PS, the tCSA and fCSA of the PS increased and the FI decreased after surgery from L2–3 to L5–S1 in patients with DLSS, which was similar to that reported in a previous study [34]. This demonstrated that compensatory hypertrophy of the PS occurs after surgery, which may play an important role in postoperative adaption and maintenance of spinal stability. The possible mechanism for PS hypertrophy might be stress transition through the anterior column of spine increasing after vertebral compression. Compensatory hypertrophy of the PS occurs at stress concentrations to maintain spinal stability [35], which may be beneficial to reduce rates of postoperative mechanical complications such as S1 screw loosening.

In this study, the S1 screw loosening rate was 36.32%. Previously, the S1 screw loosening rate was reportedly approximately 15.6–46.5% [2, 4, 7]. There are more male patients experiencing S1 screw loosening, which is similar to Wang et al. [15], Yuan et al. [36] and Kim et al.’s studies [7]. Long-segment fusion and osteoporosis are also risk factors for screw loosening [7, 37]. Additionally, in this study, the number of fused levels in the S1 screw loosening group were higher than those in the S1 screw non-loosening group (*P* < 0.05). With the limitations of the Dual-energy X-ray absorptiometry (DXA), studies recently have recommended using CT to measure the Hounsfield unit (HU) as a complementary method for assessing bone mineral density (BMD) [27, 38]. According to our results, the CT value of L1 was lower in the S1 screw loosening group than that in the control group in this study. Then, by applying these factors in the binary logistic regression analysis, we found that the male, lower CT value of L1 and longer-segment fusion were risk factors for S1 screw loosening. Recent studies also reported that paraspinal muscles played an important role in the S1 screw loosening [3, 7, 15]. Previous studies have mostly focused on the correlation between preoperative muscle status and screw loosening [7, 13, 15]. However, the relationship between the pre- and postoperative variations of PS and screw loosening has not been elucidated. Our study demonstrates, postoperative compensatory hypertrophy of the PS from L3 to S1 was observed to be lower in the S1 screw loosening group than in the controls. This is an interesting finding which reminds us of the importance on considering the postoperative changes in paraspinal muscles, especially in PS, since it is not destroyed by the posterior approach. Thus, higher compensatory hypertrophy may prove more effective in maintaining spinal stability and reducing the stress on instrumentation, decreasing the rate of S1 screw loosening.

PS contributes a lot to the spinal stabilization [35], and given the strong correlation between postoperative changes in PS and S1 screw loosening revealed in our data,, we included postoperative hypertrophy of the PS from L3 to S1, age, gender, CT value of L1, fused levels, and L5–S1 intervertebral fusion [7, 39] into the regression model for predicting screw loosening. The results demonstrated that postoperative functional PS hypertrophy might serve as a protective factor for S1 screw loosening in patients with DLSS undergoing short-segment fusion. According to the Youden index of the ROC curve for increased postoperative PS and S1 screw loosening, the threshold was found to be 1.075. While previous studies have emphasized the importance of MF and erector spinae (ES) in screw loosening [7, 15], there has been limited research focusing on the role of the PS. This study was the first to explore the relationship between pre- and postoperative variations in the PS and S1 screw loosening. Our findings suggested that postoperative hypertrophy of the PS might have a protective effect on S1 screw loosening. Thus, greater emphasis should be placed on postoperative rehabilitation exercises involving the PS, rather than focusing only on the preoperative paraspinal muscle evaluation to reduce the rates of complications. Future study could explore the surgical outcomes and rates of complications in patients after specific muscle training for PS [40–42].

However, this study had several limitations. First, this was a retrospective study and one-institution study, which was inevitably subject to selection bias. In the future, a prospective study with a larger sample size will be necessary to validate these conclusions. Second, the fCSA measurement was conducted manually and subjectively. Although previous reports have validated the measurement’s reliability [3, 15, 43], further confirmatory studies are warranted [3]. Finally, a longer follow-up period will help to better understand the clinical outcomes of the patients.

## Conclusions

In conclusion, the CSA and fCSA of the postoperative PS compared with the preoperative muscle at the same level increased and the FI decreased from L2 to S1 in patients with DLSS after posterior lumbar fusion surgery. Furthermore, the postoperative compensatory hypertrophy of the PS was more pronounced in the S1 screw non-loosening group than in the S1 screw loosening group. Male, lower CT value of L1 and longer-segment fusion were independent risk factors for S1 screw loosening. Postoperative hypertrophy of the PS at L3-S1 was determined to be a protective factor for S1 screw loosening. Finally, it is crucial to emphasize the importance of tailored rehabilitation exercises targeting the paraspinal muscles after spinal surgery, especially for patients with slight variations in the postoperative PS size. We propose a cutoff value of fCSA < 1.075 as a screening criterion for identifying such patients.

## Data Availability

The datasets used and analysed during the current study are available from the corresponding author on reasonable request.

## References

[1] Sabnis AB, Chamoli U, Diwan AD (2018). Is L5-S1 motion segment different from the rest? A radiographic kinematic assessment of 72 patients with chronic low back pain. Eur Spine J.

[2] Pihlajämaki H, Myllynen P, Böstman O (1997). Complications of transpedicular lumbosacral fixation for non-traumatic disorders. J Bone Joint Surg Br.

[3] Leng J, Han G, Zeng Y (2020). The Effect of Paraspinal Muscle Degeneration on Distal Pedicle Screw Loosening following corrective surgery for degenerative lumbar scoliosis. Spine (Phila Pa 1976).

[4] Finger T, Bayerl S, Onken J (2014). Sacropelvic fixation versus fusion to the sacrum for spondylodesis in multilevel degenerative spine disease. Eur Spine J.

[5] Birknes JK, White AP, Albert TJ (2008). Adult degenerative scoliosis: a review. Neurosurgery.

[6] Kim YJ, Bridwell KH, Lenke LG (2006). Pseudarthrosis in long adult spinal deformity instrumentation and fusion to the sacrum: prevalence and risk factor analysis of 144 cases. Spine (Phila Pa 1976).

[7] Kim JB, Park SW, Lee YS (2015). The effects of Spinopelvic Parameters and paraspinal muscle degeneration on S1 screw loosening. J Korean Neurosurg Soc.

[8] Soshi S, Shiba R, Kondo H (1991). An experimental study on transpedicular screw fixation in relation to osteoporosis of the lumbar spine. Spine (Phila Pa 1976).

[9] Xu F, Zhou S, Zou D (2022). The relationship between S1 screw loosening and postoperative outcome in patients with degenerative lumbar scoliosis. BMC Musculoskelet Disord.

[10] Shafaq N, Suzuki A, Matsumura A (2012). Asymmetric degeneration of paravertebral muscles in patients with degenerative lumbar scoliosis. Spine (Phila Pa 1976).

[11] Sun D, Liu P, Cheng J (2017). Correlation between intervertebral disc degeneration, paraspinal muscle atrophy, and lumbar facet joints degeneration in patients with lumbar disc herniation. BMC Musculoskelet Disord.

[12] Zotti MGT, Boas FV, Clifton T (2017). Does pre-operative magnetic resonance imaging of the lumbar multifidus muscle predict clinical outcomes following lumbar spinal decompression for symptomatic spinal stenosis?. Eur Spine J.

[13] Gengyu H, Jinyue D, Chunjie G (2022). The predictive value of preoperative paraspinal muscle morphometry on complications after lumbar surgery: a systematic review. Eur Spine J.

[14] Han G, Zhou S, Qiu W (2023). Role of the Paraspinal Muscles in the Sagittal Imbalance Cascade: the effects of their endurance and of their morphology on Sagittal Spinopelvic Alignment. J Bone Joint Surg Am.

[15] Wang W, Li W, Chen Z (2021). Risk factors for screw loosening in patients with adult degenerative scoliosis: the importance of paraspinal muscle degeneration. J Orthop Surg Res.

[16] Strube P, Putzier M, Streitparth F (2016). Postoperative posterior lumbar muscle changes and their relationship to segmental motion preservation or restriction: a randomized prospective study. J Neurosurg Spine.

[17] Lewis JS, Hewitt JS, Billington L (2005). A randomized clinical trial comparing two physiotherapy interventions for chronic low back pain. Spine (Phila Pa 1976).

[18] Niemistö L, Lahtinen-Suopanki T, Rissanen P (2003). A randomized trial of combined manipulation, stabilizing exercises, and physician consultation compared to physician consultation alone for chronic low back pain. Spine (Phila Pa 1976).

[19] Sun Z, Wang Y, Ji S (2015). Computer-aided analysis with Image J for quantitatively assessing psoriatic lesion area. Skin Res Technol.

[20] Takayama K, Kita T, Nakamura H (2016). New predictive index for lumbar paraspinal muscle degeneration Associated with Aging. Spine (Phila Pa 1976).

[21] Yu B, Jiang K, Li X (2017). Correlation of the features of the lumbar multifidus muscle with Facet Joint Osteoarthritis. Orthopedics.

[22] Kim JY, Ryu DS, Paik HK (2016). Paraspinal muscle, facet joint, and disc problems: risk factors for adjacent segment degeneration after lumbar fusion. Spine J.

[23] Han G, Zou D, Li X (2022). Can fat infiltration in the multifidus muscle be a predictor of postoperative symptoms and complications in patients undergoing lumbar fusion for degenerative lumbar spinal stenosis? A case-control study. J Orthop Surg Res.

[24] Hyun SJ, Kim YJ, Rhim SC (2016). Patients with proximal junctional kyphosis after stopping at thoracolumbar junction have lower muscularity, fatty degeneration at the thoracolumbar area. Spine J.

[25] Wu JC, Huang WC, Tsai HW (2011). Pedicle screw loosening in dynamic stabilization: incidence, risk, and outcome in 126 patients. Neurosurg Focus.

[26] Sandén B, Olerud C, Petrén-Mallmin M (2004). The significance of radiolucent zones surrounding pedicle screws. Definition of screw loosening in spinal instrumentation. J Bone Joint Surg Br.

[27] Zou D, Jiang S, Zhou S (2020). Prevalence of osteoporosis in patients undergoing lumbar Fusion for lumbar degenerative diseases: a combination of DXA and Hounsfield units. Spine (Phila Pa 1976).

[28] Zou D, Li W, Deng C (2019). The use of CT Hounsfield unit values to identify the undiagnosed spinal osteoporosis in patients with lumbar degenerative diseases. Eur Spine J.

[29] Chaffin DB, Redfern MS, Erig M (1990). Lumbar muscle size and locations from CT scans of 96 women of age 40 to 63 years. Clin Biomech (Bristol Avon).

[30] Danneels LA, Vanderstraeten GG, Cambier DC (2000). CT imaging of trunk muscles in chronic low back pain patients and healthy control subjects. Eur Spine J.

[31] Choi MK, Kim SB, Park BJ (2016). Do trunk muscles affect the lumbar Interbody Fusion Rate? Correlation of Trunk Muscle Cross Sectional Area and Fusion Rates after posterior lumbar Interbody Fusion using stand-alone cage. J Korean Neurosurg Soc.

[32] Verla T, Adogwa O, Elsamadicy A (2016). Effects of Psoas muscle thickness on outcomes of lumbar Fusion surgery. World Neurosurg.

[33] Roussouly P, Gollogly S, Berthonnaud E (2005). Classification of the normal variation in the sagittal alignment of the human lumbar spine and pelvis in the standing position. Spine (Phila Pa 1976).

[34] Lin GX, Ma YM, Xiao YC (2021). The effect of posterior lumbar dynamic fixation and intervertebral fusion on paraspinal muscles. BMC Musculoskelet Disord.

[35] Jorgensson A (1993). The iliopsoas muscle and the lumbar spine. Aust J Physiother.

[36] Yuan L, Zhang X, Zeng Y (2023). Incidence, risk, and Outcome of Pedicle Screw Loosening in degenerative lumbar scoliosis patients undergoing Long-Segment Fusion. Global Spine J.

[37] Bokov A, Bulkin A, Aleynik A (2019). Pedicle screws loosening in patients with degenerative diseases of the lumbar spine: potential risk factors and relative contribution. Global Spine J.

[38] Alacreu E, Moratal D, Arana E (2017). Opportunistic screening for osteoporosis by routine CT in Southern Europe. Osteoporos Int.

[39] Yu BS, Zhuang XM, Zheng ZM (2010). Biomechanical comparison of 4 fixation techniques of sacral pedicle screw in osteoporotic condition. J Spinal Disord Tech.

[40] Rackwitz B, de Bie R, Limm H (2006). Segmental stabilizing exercises and low back pain. What is the evidence? A systematic review of randomized controlled trials. Clin Rehabil.

[41] Barr KP, Griggs M, Cadby T (2007). Lumbar stabilization: a review of core concepts and current literature, part 2. Am J Phys Med Rehabil.

[42] Hou X, Hu H, Kong C (2023). Correlation of psoas major muscle morphology with function and clinical symptoms in patients with symptomatic multilevel lumbar spinal stenosis. J Orthop Surg Res.

[43] Fan S, Hu Z, Zhao F (2010). Multifidus muscle changes and clinical effects of one-level posterior lumbar interbody fusion: minimally invasive procedure versus conventional open approach. Eur Spine J.

